# St. George’s Hospital

**Published:** 1852-11

**Authors:** 


St. George's Hospital. *Physicians*—Drs. Wilson, Nairne, Page, and Bence Jones. *Assistant Physicians*—Drs. Pitman and Fuller. *Surgeons*—Messrs. Keate, Cæsar Hawkins, Cutler, and Tatum. *Assistant Surgeons*—Messrs. Johnson and Hewett.

Medical and Surgical School. *Winter Session.*—Anatomy, Genera!, and Physiology, Mr. A. Johnson. Anatomy, Descriptive and Surgical, Messrs. Hewett and Pollock. Chemistry, Mr. Noad. Medicine, Drs. Naird and Page. Surgery, Mr. Tatum. *Summer Session.*—Materia Medica, Dr. Pitman. Midwifery, Dr. R. Lee. Botany, Mr. Henfrey. Medical Jurisprudence, Dr. Fuller and Mr. H. Johnson.

